# Bioactive Substances in Sheep’s Milk and Related Products as Components of Functional Foods

**DOI:** 10.3390/molecules31162848

**Published:** 2026-08-14

**Authors:** Zuzanna Flis, Jarosław Wieczorek, Marek Sady, Edyta Molik

**Affiliations:** 1 Department of Animal Biotechnology, Faculty of Animal Sciences, University of Agriculture in Krakow, A. Mickiewicz 24/28 Av., 30-059 Krakow, Poland; 2 Department of Diagnostics and Clinical Sciences, Faculty of Veterinary Medicine, University of Agriculture in Krakow, A. Mickiewicz 24/28 Av., 30-059 Krakow, Poland; jaroslaw.wieczorek@urk.edu.pl; 3 Department of Animal Products Processing, Faculty of Food Technology, University of Agriculture in Krakow, Balicka 122, Av., 30-149 Krakow, Poland; marek.sady@urk.edu.pl

**Keywords:** sheep milk, fermented products, bioactive compounds, functional food ingredients

## Abstract

Sheep milk and its fermented products are increasingly recognized as valuable functional foods due to their high nutritional value and the presence of biologically active compounds. The aim of this review was to summarize current knowledge on selected bioactive components of sheep milk and fermented sheep milk products, with particular emphasis on their potential application as functional food ingredients and the impact of technological processes on their biological properties. The review focuses on yogurts, kefirs, and cheeses, highlighting key bioactive compounds, including peptides, lactoferrin, conjugated linoleic acid (CLA), polar lipids, orotic acid, and probiotic microorganisms. Fermentation and maturation processes contribute to the formation and transformation of bioactive compounds, which may exhibit antioxidant, antimicrobial, antihypertensive, and immunomodulatory activities, as well as support gut health and metabolic regulation. The functional potential of sheep milk products depends on milk composition, microbial activity, and processing conditions. Despite promising findings, their wider application remains limited due to seasonal availability, low production scale, and insufficient knowledge regarding bioavailability and physiological effects. Further in vivo and clinical studies are required to confirm the health-promoting potential and practical applications of fermented sheep milk products as functional food ingredients.

## 1. Introduction

In many regions of Europe, the production of sheep milk and dairy products is an important element of traditional agriculture. It primarily relies on extensive grazing and local breeding practices that have been passed down through generations. These animal production systems are particularly characteristic of mountainous areas, allowing sheep to be kept in low-input systems that utilize natural feed resources and seasonal grazing [1]. Sheep farming in traditional extensive systems serves not only an economic but also a cultural and environmental function. It contributes to the maintenance of the mountain landscape, the preservation of local traditions, and the promotion of pastoral culture [2]. Sheep milk obtained in extensive systems is strongly linked to traditional processing methods, which are based on artisanal cheese-making practices passed down within local pastoral communities [3].

**Figure 1:**
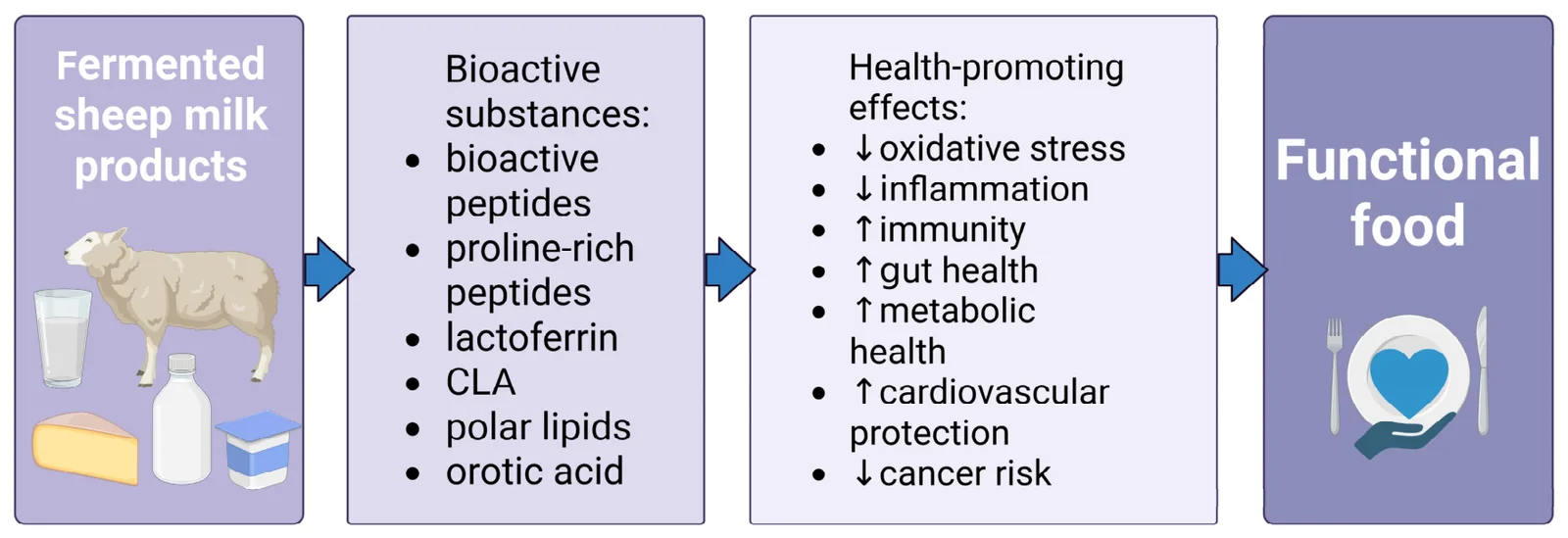
Possible use of bioactive substances of sheep milk and its products as functional food. In the schematic, downward arrows indicate a decrease in the effect, whereas upward arrows indicate an enhancement of the effect. Created in BioRender. Flis, Z. (2026) https://BioRender.com/9fox6ui.

In Europe, sheep’s milk is an important raw material for the production of regional cheeses, especially in Mediterranean and mountainous areas [4]. The specific conditions of sheep farming, seasonal grazing, and artisanal production methods contribute to the unique sensory, qualitative, and health-promoting properties of these products [4]. Growing consumer interest in fermented sheep’s milk products is driven by the demand for regional, minimally processed foods with high nutritional value, particularly within the functional food sector [5]. Additionally, gastronomic tourism supports the promotion of traditional dairy products, enhancing their cultural and economic importance in production regions [6,7]. The functional potential of sheep’s milk products is linked to their bioactive compounds and health-promoting effects, contributing to the expanding market for fermented dairy foods [8]. The potential of bioactive compounds present in sheep milk and its products for use in functional foods is summarized in Figure 1.

**Figure 2:**
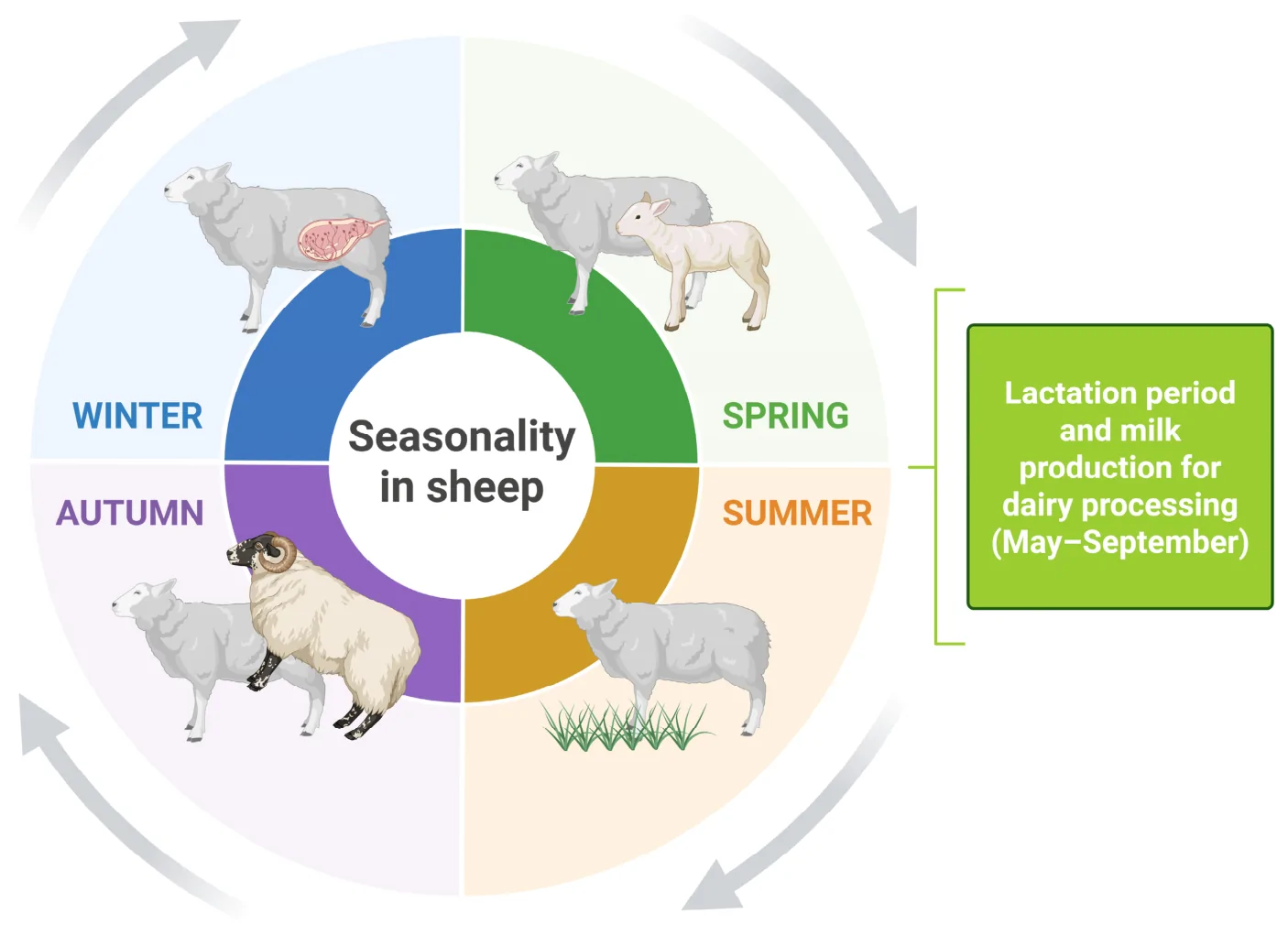
Seasonal pattern of sheep milk production in extensive farming systems. Created in BioRender.com. Flis, Z. (2026) https://BioRender.com/kzis1c2.

Despite its high nutritional value and the presence of numerous bioactive compounds, sheep milk production is characterized by pronounced seasonality, which remains one of the major factors limiting the wider utilization of this raw material. The seasonal pattern of milk production is closely related to the reproductive cycle of sheep and the grazing-based husbandry system, making year-round availability difficult to achieve [9]. The seasonality of sheep milk production is presented in Figure 2.

At the same time, sheep milk and products derived from it are a rich source of bioactive compounds, the functional potential of which is increasingly being investigated. Compared to the milk of other animal species, sheep milk is characterized by a favorable nutrient profile and a higher content of selected bioactive compounds, such as proteins, peptides, conjugated linoleic acid (CLA), and lactoferrin [10]. However, it should be emphasized that current knowledge focuses primarily on characterizing the chemical composition and potential biological activity of individual components, while issues related to their bioavailability, stability during processing, and the possibility of practical application in the design of functional foods still require further investigation.

Therefore, the aim of this review is to analyze the potential of sheep milk and fermented products as a source of natural bioactive substances, with particular emphasis on bioactive peptides, lactoferrin, conjugated linoleic acid (CLA), polar lipids, orotic acid, and probiotic microorganisms. The review also discusses the impact of fermentation and maturation processes on the formation and transformation of bioactive compounds, as well as technological limitations and future directions related to the use of sheep milk as a functional food ingredient.

## 2. Processing of Sheep Milk

### 2.1. Fermentation and Ripening Processes

Milk is widely used in the production of fermented dairy products, such as yogurt, kefir, and various cheeses. These products constitute an important segment of traditional and functional dairy foods. Fermentation and ripening processes play a key role in the production of dairy products, during which intensive biochemical changes occur, including those resulting from the activity of starter microorganisms [11,12].

### 2.2. Biochemical Transformations During Sheep Milk Processing

The activity of lactic acid bacteria and other microorganisms leads to the degradation of proteins, lipids, and lactose. This results in the formation of a wide spectrum of metabolites with diverse chemical structures and properties. In particular, the proteolysis of caseins and whey proteins leads to the release of bioactive peptides, which may exhibit antioxidant, antihypertensive, and immunomodulatory effects [13]. During fermentation and ripening of cheeses, the lipid profile also undergoes intensive modification, including the formation of free fatty acids and their derivatives. These processes influence the sensory characteristics of products and their potential biological activity [14].

### 2.3. Microbial Influence on Quality Characteristics of Sheep Milk Products

Changes in the microbiological structure are observed, with lactic acid bacteria strains primarily influencing both the fermentation process and the final product quality [12]. These processes result in dairy products with a diverse nutritional and sensory profile, which is directly related to the degree of proteolysis, lipolysis, and enzymatic activity of the microflora [12,15].

## 3. Sheep Milk Products

### 3.1. Nutritional Characteristics and Functional Potential of Sheep Milk Products

Compared to the milk of other ruminants, including the most commonly consumed cow’s milk, sheep milk is characterized by a higher content of protein, fat, vitamins, and minerals, which contributes to a higher concentration of bioactive compounds and higher nutritional value [16]. Furthermore, its favorable chemical composition, including high dry matter, protein, and fat content, makes sheep milk widely used in the production of fermented dairy products such as yogurt, kefir, and various cheeses. Each of these product groups is characterized by a different production technology and degree of processing, which influences their structure, sensory properties, and physicochemical and bioactive stability [12,17].

### 3.2. Fermented Sheep Milk Products: Yogurt and Kefir

Sheep milk yogurt is produced through controlled fermentation by lactic acid bacteria, primarily from the genera *Lactobacillus* and *Streptococcus*. These microorganisms are responsible for lowering pH, coagulating proteins, and shaping the product’s texture [18,19]. Compared with cow’s milk yogurt, sheep milk products are characterized by a higher dry matter content, which contributes to their more compact structure and more intense sensory profile. Kefir is a product of mixed fermentation involving lactic acid bacteria, acetic acid bacteria, and yeast. The complexity of this microflora leads to more complex biochemical transformations and the formation of compounds responsible for its characteristic slightly carbonated consistency [20].

### 3.3. Sheep Milk Cheeses: Traditional Products and Bioactive Potential

Sheep milk cheeses constitute a particularly diverse group of fermented products. They include fresh cheeses, cottage cheeses, hard cheeses, and long-ripened cheeses, as well as cheeses enriched with flavorings. The most well-known cheeses include those from the Mediterranean region, such as Greek feta and kefalotyri, and Italian sheep milk cheeses, including *Pecorino romano* and *Pecorino sardo* [3,14]. In Poland, examples of such products include Podhale cheeses, including oscypek, bryndza, and bundz, which are produced using traditional shepherding methods and milk from local sheep breeds [1,21]. These cheeses are produced by a combination of enzymatic coagulation and long-term ripening, during which intensive protein and fat transformations occur [14,15]. Different types of cheese differ in production technology, ripening time, and sensory profile. In particular, mature cheeses are characterized by advanced levels of proteolysis and lipolysis, which influence their texture, aroma, and flavor [14]. This wide variation stems primarily from their artisanal nature, where local recipes, environmental conditions, and raw material availability determine the final nutritional and bioactive profile of the product [22].

### 3.4. Bioactive Compounds and Functional Potential of Fermented Sheep Milk Products

Sheep milk and its derived products contain various bioactive compounds associated with their nutritional and functional characteristics. In fermented products, the composition and properties of these compounds may be influenced by microbial activity occurring during fermentation and ripening. The main selected bioactive compounds identified in sheep milk and fermented sheep milk products, together with their potential functional significance, are summarized in Table 1.

The functional potential of fermented sheep milk products depends not only on the initial composition of milk but also on fermentation microorganisms, ripening conditions and processing technology.

## 4. Selected Bioactive Compounds of Sheep Milk and Its Products

Sheep milk contains a wide range of bioactive compounds that contribute to its nutritional and functional value. The following sections present the major selected bioactive constituents identified in sheep milk and dairy products, while Table 2 provides a comparison of selected bioactive components in sheep, cow, and goat milk.

The functional potential of fermented sheep milk products depends not only on the initial composition of milk but also on the type and activity of fermentation microorganisms, ripening conditions, and processing technology. These factors influence the formation, transformation, and stability of bioactive compounds, thereby affecting the nutritional value and potential health-promoting properties of the final products.

### 4.1. Bioactive Peptides

Fermented sheep milk products, such as kefir, yogurt, and cheese, are significant sources of bioactive components formed through the intensive proteolysis of milk proteins, including α-lactalbumin, lactoferrin, and proline-rich peptides. In the case of sheep milk kefir, peptidomic analysis revealed the identification of over 1900 peptides derived mainly from casein, 78 of which showed potential biological activity, including antioxidant and immunomodulatory effects, and their number increased with fermentation time [22]. For this reason, interest in these products as potential nutraceuticals has increased in recent years [28]. Increased proteolysis is observed in sheep milk yogurts during storage, leading to the formation of bioactive peptides with antihypertensive properties, mainly β-casein derivatives [29]. In sheep cheeses, the ripening process promotes the accumulation of bioactive peptides, including angiotensin-converting enzyme (ACE) inhibitors and antioxidant compounds produced by microbial enzymes and milk proteases [30].

Compared to cow’s milk, sheep milk contains significant amounts of proline (Table 2), which is an important amino acid present in its protein fraction [24]. Proline is one of the key amino acids involved in the biosynthesis of collagen, which is responsible for the proper structure and function of connective tissue, skin, cartilage, and bones [31]. Furthermore, proline and peptides containing this amino acid may exhibit potential biological activity, including antioxidant properties and participation in the regulation of metabolic processes [32,33]. Therefore, the presence of proline and proline-containing peptides in fermented dairy products may be one of the components contributing to their functional potential.

During fermentation and ripening of dairy products, milk protein transformations occur, leading to the release of free amino acids and short peptides. Proline may remain part of the product’s amino acid profile or be released during proteolytic processes, influencing both the biochemical and sensory properties of fermented products [34]. Due to its characteristic flavor properties associated with a mild, slightly sweet taste, its presence may contribute to shaping the final flavor profile of the product [34].

In sheep milk cheeses, ripening is associated with intensive casein proteolysis, which leads to a gradual increase in the content of free amino acids in the cheese matrix. Due to its presence in the structure of milk proteins, proline is one of the amino acids detected in the free amino acid profile of ripening cheeses and may accumulate during this process [35]. Studies conducted on cheeses, including Pecorino, indicate that changes occurring during ripening affect the final amino acid composition of the product and its sensory properties [36].

The importance of fermentation processes in proline metabolism is also confirmed by studies on the traditional fermented dairy product Gioddu, which demonstrated differences in its metabolomic profile compared with sheep milk. The increased content of selected amino acids, including proline, was associated with the activity of fermentative microorganisms and the transformation of milk proteins during fermentation [34].

Sheep milk is one of the richest sources of lactoferrin among mammalian milks, and its content is higher than that in cow’s and goat’s milk (Table 2), which highlights its high functional value [23,37]. In recent years, the multifaceted effects of lactoferrin have been confirmed, demonstrating its strong health-promoting properties. Lactoferrin exhibits effective antibacterial and antiviral effects [38]. It also exhibits antifungal and antiparasitic properties [39,40]. Lactoferrin acts through mechanisms such as iron sequestration, disruption of microbial cell membranes, inhibition of microbial adhesion, and disruption of host–pathogen interactions [41]. It also has immunomodulatory effects, including inhibiting pathogen adhesion, limiting pathogen invasion, and modulating the host’s inflammatory response [42]. Furthermore, lactoferrin has been shown to have antioxidant, anti-inflammatory, and anticancer effects [41,43,44].

In dairy products such as yogurt, kefir, and cheese, the content and activity of lactoferrin can change as a result of technological and fermentation processes, but research on its stability and metabolism in these products remains limited. Fermentation of sheep milk leads to intensive transformations of milk proteins, including partial proteolysis and the release of bioactive peptide fragments [22]. Studies conducted on sheep milk kefir have shown that with increasing fermentation time, the proteolytic activity of microorganisms increases, as does the number of identified peptides with potential biological activity [22]. However, it should be emphasized that this activity depends on the type of peptide sequences released and the source of the protein from which they are derived.

In ripened sheep milk cheeses, the proteolytic process is advanced and leads to the gradual transformation of the protein fraction. As a result, numerous peptides and free amino acids are formed, which can affect the functional properties of the product. The intensity of these transformations depends on factors such as ripening time, the activity of milk-derived enzymes, and the proteolytic activity of microorganisms present in the cheese [45,46].

Available research also indicates that lactoferrin can be used as a fortifying ingredient in dairy products. In a study on cheddar cheese, the addition of lactoferrin did not significantly affect the product’s composition, fatty acid profile, or sensory characteristics, confirming its potential for use without compromising product quality [47]. Similar observations were made in yogurt, where the addition of lactoferrin did not significantly affect the product’s physicochemical and microbiological properties or the course of lactic acid fermentation. It was also shown that lactoferrin maintained high stability during storage [48]. Due to their relatively high lactoferrin content, sheep milk products may represent an interesting avenue for functional food development. However, further research is needed on the stability, bioavailability, and biological activity of lactoferrin in these products.

Sheep milk whey is a significant source of bioactive proteins. Compared with bovine whey, it is characterized by a higher content of biologically active components, such as lactoferrin, α-lactalbumin, and β-lactoglobulin [49]. During the cheesemaking process, most casein coagulates and forms the curd, while whey proteins, including lactoferrin, remain in the liquid phase, making whey an important carrier of functional compounds [14,49,50]. Lactoferrin present in whey exhibits strong antibacterial, antiviral, and immunomodulatory properties resulting from its ability to bind iron, inhibit pathogen adhesion, and modulate the inflammatory response [42]. As a result, sheep whey is now perceived not as technological waste but as a valuable raw material used in the production of functional foods [49]. Sheep whey plays a particularly important role in traditional Polish dairy products from mountainous regions. An example is żętyca, a beverage obtained from whey left over from the production of oscypek and bundz [51]. Its preparation process involves heating the whey, which transforms whey proteins, followed by natural fermentation carried out by the product’s microflora [51]. The production of traditional sheep cheeses, such as bundz, is also closely linked to the formation of whey, which can be an additional source of valuable nutrients [51]. The growing interest in sheep whey stems not only from its nutritional value but also from its potential use as a source of bioactive peptides, such as lactoferrin, which exhibit potential antioxidant, antibacterial, and immunomodulatory properties. Therefore, products made from sheep whey, including traditional whey products, may be interesting components of functional foods; however, research on their composition and biological activity remains limited.

One potential avenue for increasing the use of sheep whey as a functional raw material is to combine traditional processing methods with modern membrane separation technologies. Ultrafiltration can enable the concentration of whey protein fractions, including lactoferrin, and improve the recovery and standardization of bioactive components [52,53]. This approach could contribute to the development of new functional food ingredients while reducing losses of valuable components generated during traditional sheep cheese production [54]. In the longer term, membrane technologies may provide a tool for more effective utilization of dairy by-products as sources of valuable bioactive compounds.

Despite numerous studies confirming the biological activity of milk protein-derived peptides in vitro, their in vivo efficacy remains a subject of investigation. The bioavailability of peptides depends on their resistance to digestion in the gastrointestinal tract, their potential for absorption, and their metabolic stability after ingestion [55]. Therefore, not all peptides exhibiting high biological activity in laboratory studies retain the same effects after human ingestion, indicating the need for further research on their bioavailability and clinical efficacy [56].

An important direction for further research is to optimize the fermentation process to increase the release of peptides with angiotensin-converting enzyme (ACE)-inhibitory activity [55]. Selecting appropriate lactic acid bacteria strains with high proteolytic activity and controlling fermentation conditions may promote the formation of peptides with greater biological potential [57]. In the case of sheep milk, due to its high protein content and intensive proteolytic transformations occurring during fermentation and ripening, modifying the technological process may be an effective strategy to increase the release of peptides with the desired activity.

### 4.2. Fatty Acids

Sheep milk and products derived from it are among the richest natural sources of conjugated linoleic acid (CLA). The CLA content in sheep milk is higher than that in cow’s milk (Table 2) [23]. The most important biologically active CLA isomers are cis-9, trans-11 and trans-10, cis-12, which exhibit distinct but complementary metabolic activities. The cis-9, trans-11 isomer is primarily associated with potential anticancer, antiatherosclerotic, and anti-inflammatory effects through the modulation of processes related to the inflammatory response and lipid metabolism [58,59]. The trans-10, cis-12 isomer of CLA, on the other hand, exhibits stronger lipolytic and anti-obesity properties by modulating lipid metabolism, regulating the activity of enzymes involved in fat synthesis and storage, and inhibiting triglyceride accumulation in adipocytes [58,59]. Furthermore, CLA is associated with the regulation of metabolic pathways dependent on peroxisome proliferator-activated receptors, particularly PPARα, which participate in the control of lipid metabolism, the inflammatory response, and energy homeostasis [60].

Studies have shown that sheep milk and its products, including kefir and cheese, contain significantly higher concentrations of CLA compared with cow’s milk products [61,62]. Kefirs obtained from sheep milk were shown to have lower atherogenicity and thrombogenicity indices and a more favorable omega-6 to omega-3 fatty acid ratio [61]. This suggests potentially more beneficial nutritional properties of sheep milk in the context of cardiovascular disease prevention. Traditional Greek yogurts obtained from sheep milk are valuable sources of bioactive components of milk fat, including CLA [62]. Studies have shown that the CLA content in sheep milk yogurts ranged from 0.405 to 1.250 g/100 g of fat, and its level depended on factors such as the origin of the product and animal husbandry conditions [62]. A particularly high CLA content was observed in traditional Greek yogurts from mountainous regions, which may be related to the greater proportion of natural pasture-based diets of sheep [62]. Dairy products obtained from sheep milk, especially those from traditional mountain production systems, can be valuable sources of CLA. Studies conducted on traditional Bulgarian sheep milk products demonstrated high CLA content. The highest CLA content was recorded in white brined cheese (35.6 mg/g fat), followed by sheep milk yogurt (29.5 mg/g fat), while yellow sheep cheese had a CLA content of 21.8 mg/g fat. These results indicate that the type of dairy product and the processing technology used can significantly influence the level of this bioactive component [63].

Importantly, the CLA content in fermented products can change during refrigerated storage. In the case of sheep milk yogurt, an increase in CLA content was demonstrated during 14-day storage at 5 °C [51]. Furthermore, a decrease in CLA content was observed in cow’s milk yogurts, indicating a significant effect of milk type on the stability and metabolism of fatty acids over time [62]. In summary, CLA present in sheep milk products has several potential health-promoting effects, including improving blood lipid profiles, exerting anti-inflammatory effects, modulating the immune response, and influencing energy metabolism and gut microbiota composition, making it an important functional dietary component [64].

Polar lipids are a milk lipid fraction with significant biological value (Table 2) [65]. They are located primarily in the membrane surrounding milk fat globules (MFGM) [66]. The most important polar lipids include phospholipids, such as phosphatidylcholine, phosphatidylethanolamine, and phosphatidylserine, as well as sphingolipids, particularly sphingomyelin [66,67]. These compounds play a key role in stabilizing fat emulsions and shaping the functional properties of dairy products [66,68]. A growing body of research also indicates their possible health-promoting effects, including modulation of gut microbiota, lipid metabolism, immune response, and neurological function [66,68,69].

Fermentation processes significantly modify the composition and activity of polar lipids in dairy products. In the case of sheep milk yogurt, changes in the phospholipid profile of MFGM have been observed, including an increased content of phosphatidylethanolamine, phosphatidylcholine, and sphingomyelin [70]. Additionally, Lordan et al. (2020) observed an increase in biological activity related to antiplatelet and antithrombotic effects, including through the modulation of platelet-activating factor (PAF) and thrombin systems [70].

The most pronounced changes in the fat emulsion structure occur in cheeses, particularly sheep milk and ripened cheeses, which are significant sources of polar lipids bound to MFGM fragments. Milk processing, including fermentation, leads to the release of polar lipids from MFGM into the product matrix, increasing their bioavailability. During the ripening and fermentation of sheep milk products, significant modifications occur in the phospholipid profile, along with an increase in their bioactivity, including anti-PAF and antithrombotic activity [71]. Sheep cheeses such as Ladotyri and Kefalotyri have been shown to exhibit antiatherosclerotic and antithrombotic effects, which are associated with the presence of active PAF inhibitors [71,72].

### 4.3. Orotic Acid

Orotic acid is an intermediate metabolite in the pyrimidine biosynthesis pathway, playing an important role in nucleotide synthesis and the regulation of cellular proliferation and metabolism [25,73]. In ruminant milk, particularly sheep milk, its content is significantly higher than in cow’s and human milk, reaching average values of approximately 200–400 mg/L and 25–70 mg/L, respectively (Table 2) [25]. Orotic acid content in milk may depend on factors such as the stage of lactation, breed, and feeding conditions [25,74].

Orotic acid content in cheeses depends on the amount of soluble whey solids, the degree of fermentation, and ripening time [75]. The orotic acid profile has been found to vary depending on the cheese variety and is important for the flavor of some cheese products [76]. Sheep milk and its products, especially ripened cheeses, may be important sources of orotic acid in the human diet due to its high health-promoting potential [77,78]. However, Manolaki et al. (2006) demonstrated that the orotic acid content in feta cheese made from sheep milk decreases significantly during the first 2 days of ripening, which is related to its utilization by lactic acid bacteria as a growth factor and its metabolic transformation under reduced pH conditions [76]. In the case of kefir, the orotic acid content was shown to decrease slightly during the fermentation process [79]. On the other hand, another study by Güzel-Seydim et al. (2000) showed that the concentrations of orotic and citric acids slightly increased during refrigerated storage of kefir [79]. Kefir, as a product of mixed fermentation involving lactic acid bacteria and yeast, undergoes intensive transformation of low-molecular-weight metabolites, including the accumulation of organic acids. In yogurt, orotic acid can be partially degraded by starter cultures. In ripened sheep milk cheeses, however, orotic acid exhibits greater stability and potential accumulation, which results from limited microbial activity in the later stages of ripening and a concentration effect caused by water loss and its integration into the protein-lipid matrix [77]. Due to the variable dynamics of orotic acid in various fermented products, further research is needed on the impact of sheep milk processing on its content and health-promoting potential.

Orotic acid exhibits potential bioactive effects primarily related to its role in cellular metabolism and pyrimidine nucleotide biosynthesis [77,78]. As a natural intermediate metabolite of the de novo pyrimidine synthesis pathway, it participates in the formation of nucleotides necessary for DNA and RNA synthesis, which may be important for the proliferation, regeneration, and proper functioning of cells with high metabolic activity [80,81]. Its potential effects include, among others, its impact on myocardial energy metabolism and potential cardioprotective effects. Experimental studies indicate that orotic acid and its derivatives may support cardiomyocyte function by modulating energy metabolism, increasing the availability of metabolic substrates, and improving cardiac cell tolerance to ischemia and reperfusion stress [82]. Attention has also been drawn to the role of orotic acid in regulating mitochondrial metabolism and processes related to cell maturation and function. It has been shown that this compound can be utilized by erythrocytes and hepatocytes as a source of uridine in the pyrimidine recycling pathway, indicating its importance in maintaining normal nucleotide metabolism [80,81]. Furthermore, increasing attention is being paid to the potential impact of orotic acid on lipid metabolism and epithelial cell function. Studies indicate that this compound can modulate selected metabolic pathways related to the synthesis of milk components and the cellular response to oxidative stress, suggesting its role as a regulator of metabolic processes [83]. Consequently, dairy products, especially those of sheep origin, may be significant sources of orotic acid; however, its content and potential biological activity depend on many factors, such as the composition of the microflora, the fermentation process, and the processing technology used [81].

### 4.4. Probiotic Potential of Fermented Products

Fermented sheep milk products, such as yogurt, kefir, and cheese, are significant sources of live bacteria and postbiotic metabolites that can modulate gut microbiota and immune function (Table 1) [84,85]. Furthermore, components formed during fermentation, such as exopolysaccharides, bioactive peptides, and short-chain fatty acids, may exhibit anti-inflammatory and barrier-enhancing effects [85]. For this reason, fermented sheep milk products are increasingly recognized as potential functional foods supporting digestive health [84,85]. Their potential is attributed mainly to the presence of probiotic microorganisms and postbiotic metabolites. In addition, differences in milk protein composition and the reduction in lactose content during fermentation may influence the digestive tolerance of fermented sheep milk products. This may contribute to improved digestive tolerance in some individuals compared with conventional cow’s milk products. However, further clinical trials are needed to confirm their potential role in dietary strategies supporting individuals with gastrointestinal disorders such as IBS and IBD [86,87]. Studies have shown that fermented sheep milk provides a suitable matrix for probiotic bacteria (*Lacticaseibacillus casei* and *Lacticaseibacillus johnsonii*), ensuring high cell survival during simulated in vitro digestion [88]. This is related to the high protein and fat content and the buffering capacity of sheep milk, which may contribute to bacterial protection against gastric acidity and bile salts [84,88].

## 5. Challenges and Future Perspectives

Despite the growing interest in sheep milk and fermented products as sources of bioactive compounds, there are still many limitations hindering their wider use as functional food ingredients. The most important challenges include the seasonal availability of raw materials, limited production scale, lack of full standardization of technological processes, and insufficient knowledge regarding the stability, bioavailability, and physiological significance of individual bioactive components in the human body [55,56]. Despite numerous studies demonstrating the potential antioxidant, anti-microbial, antihypertensive, and immunomodulatory properties of compounds derived from sheep milk, most results have been obtained in vitro. Therefore, further in vivo and clinical studies are necessary to determine the actual importance of these components in human nutrition [55,56].

One promising approach to increasing the use of sheep milk as a functional raw material is the application of modern processing technologies that enable the stabilization of the raw material, reduce losses of valuable components, and preserve its bioactive potential. Freeze-drying can be a particularly interesting method for preserving milk and dairy products, limiting changes occurring during storage, and achieving products with an extended shelf life. Studies conducted on freeze-dried goat milk indicate that this process can preserve a similar profile of selected bioactive components compared with fresh milk, demonstrating the potential of this technology in the design of innovative dairy products [89]. Therefore, freeze-drying may be an interesting strategy for mitigating the seasonal availability of raw materials and increasing the possibilities of using sheep milk outside the production season.

At the same time, membrane separation technologies such as ultrafiltration can increase the recovery and standardization of whey protein fractions, including lactoferrin-enriched fractions, supporting the development of new functional food ingredients [90]. The advantage of these methods is their ability to selectively concentrate proteins while maintaining their biological properties, which is particularly important for compounds sensitive to high temperatures [53]. The resulting whey protein concentrates can be valuable functional ingredients for enriching dairy products and other food preparations [91].

**Figure 3:**
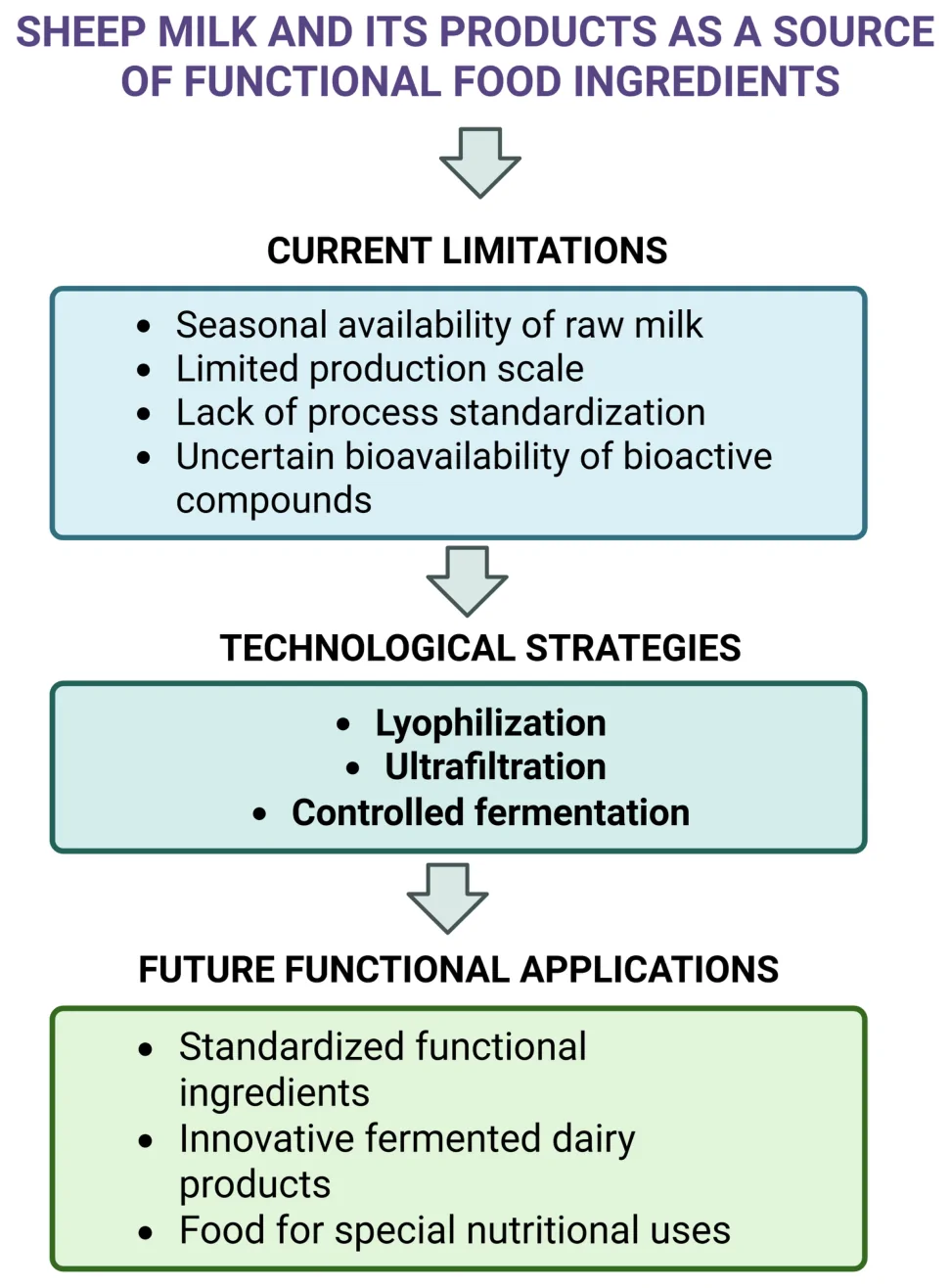
Challenges, technological strategies and future perspectives for the utilization of sheep milk as a source of functional food ingredients. Created in BioRender.com. Flis, Z. (2026) https://BioRender.com/j4dvy16.

In the future, the greatest potential may lie in the integration of several processing technologies, including raw material stabilization, selective recovery of bioactive fractions, and controlled fermentation using carefully selected microorganisms. This comprehensive approach could enable the design of products with specific functional profiles, tailored to consumer needs and specialty food applications. The technological strategies and their potential applications for the development of functional food ingredients are presented in Figure 3.

## 6. Conclusions

Sheep milk and its fermented products represent promising examples of foods with significant functional potential. They are characterized by a high content of bioactive peptides, lactoferrin, CLA, polar lipids, and microbial metabolites. Their unique composition results from both the natural properties of sheep milk and the biochemical transformations occurring during fermentation and maturation. Traditional processing methods, especially those used in mountain and artisanal production systems, contribute to the creation of products with diverse nutritional, sensory, and potentially health-promoting properties. Fermented sheep milk products can be valuable components of a functional diet due to their possible positive effects on gut microbiota, immune system modulation, lipid metabolism, and antioxidant defense. However, despite promising experimental and compositional data, further research is necessary to determine their clinical significance, the bioavailability of active substances, and potential applications in preventive medicine and nutritional therapy.

## Figures and Tables

**Table 1:** Bioactive compounds identified in fermented sheep milk products and their potential functional significance.

Fermented Sheep Milk Product	Main Bioactive Compounds	Reported Biological Activities
Sheep milk yogurt	Bioactive peptides, lactoferrin, CLA, probiotic microorganisms	Antioxidant, ACE-inhibitory, immunomodulatory and gut microbiota-supporting potential
Sheep milk kefir	Bioactive peptides, microbial metabolites, exopolysaccharides, probiotic bacteria and yeasts	Potential antioxidant, antimicrobial and immunomodulatory effects; support of intestinal microbiota
Fresh and ripened sheep milk cheeses	Bioactive peptides, CLA, polar lipids, free amino acids	Potential antioxidant, ACE-inhibitory and metabolic regulatory activities; increased bioactive peptide formation during ripening
Sheep whey products (e.g., żętyca)	Lactoferrin, whey proteins, whey-derived peptides, microbial metabolites	Potential source of functional whey proteins and bioactive compounds for functional food applications

**Table 2:** Comparison of selected bioactive compounds in sheep, cow and goat milk and their functional significance.

Bioactive Substance	Sheep Milk	Cow Milk	Goat Milk	Reported Biological Activities
Lactoferrin (g/L) [23]	0.7–0.9	0.02–0.5	0.02–0.3	Antimicrobial, antiviral, immunomodulatory and anti-inflammatory potential; important functional protein of milk
Proline in whey proteins (mg/g) [24]	102	69	67–69	Amino acid involved in collagen synthesis; proline-rich peptides may contribute to antioxidant and metabolic activity
CLA (% of total fatty acids) [23]	0.3–1.8	0.3–1.6	~0.7	Potential regulation of lipid metabolism, inflammatory response and oxidative processes
Orotic acid (mg/L) [25]	20–400	20–100	20–400	Intermediate of pyrimidine metabolism; potential role in cellular metabolism and nucleotide synthesis
Polar lipids (% of total fatty acids)	~0.39 [26]	~0.36 [27]	Not available	Components of milk fat globule membrane; associated with modulation of lipid metabolism, gut microbiota and immune function
Casein content (g/kg) [23]	48	26	30	Source of bioactive peptides released during fermentation and enzymatic hydrolysis

## Data Availability

No new data were created or analyzed in this study. Data sharing is not applicable to this article.

## Abbreviations

The following abbreviations are used in this manuscript:

CLA	Conjugated Linoleic Acid
ACE	Angiotensin-Converting Enzyme
PAF	Platelet-Activating Factor
